# Early life home microbiome and hyperactivity/inattention in school-age children

**DOI:** 10.1038/s41598-019-53527-1

**Published:** 2019-11-22

**Authors:** Lidia Casas, Anne M. Karvonen, Pirkka V. Kirjavainen, Martin Täubel, Heidi Hyytiäinen, Balamuralikrishna Jayaprakash, Irina Lehmann, Marie Standl, Juha Pekkanen, Joachim Heinrich

**Affiliations:** 1 Centre for Environment and Health – Department of Public Health and Primary Care, KU Leuven, Leuven, Belgium; 2 Environmental Health Unit, Department of Health Security, Finnish Institute for Health and Welfare, Kuopio, Finland; 3 Institue of Public Health and Clinical Nutrition, Department of Clinical Nutrition, University of Eastern Finland, Kuopio, Finland; 4 Charitè – Universitätsmedizin Berlin and Berlin Institute of Health, Berlin, Germany; 5 Department of Environmental Immunology/Core Facility Studies, Helmholtz Centre for Environmental Research – UFZ, Leipzig, Germany; 6 Institute of Epidemiology, Helmholtz Zentrum München - German Research Center for Environmental Health, Neuherberg, Germany; 7 Department of Public Health, University of Helsinki, Helsinki, Finland; 8 Institute and Outpatient Clinic for Occupational, Social and Environmental Medicine, University Hospital Munich, Ludwig Maximillians University Munich, Member of German Center for Lung Research (DZL), Munich, Germany

**Keywords:** Microbial communities, Neurology, Risk factors

## Abstract

This study evaluates the association between indoor microbial diversity early in life and hyperactivity/inattention symptoms in children at ages 10 and 15 years.A random sample enriched with subjects with hyperactivity/inattention at age 15 years was selected from the German LISA birth cohort. Bedroom floor dust was collected at age 3 months and 4 bacterial and fungal diversity measures [number of observed operational taxonomic units (OTUs), Chao1, Shannon and Simpson indices] were calculated from Illumina MiSeq sequencing data. Hyperactivity/inattention was based on the Strengths and Difficulties Questionnaire at ages 10 and 15 (cut-off ≥7). Adjusted associations between 4 diversity measures in tertiles and hyperactivity/inattention were investigated with weighted and survey logistic regression models. We included 226 individuals with information on microbial diversity and hyperactivity/inattention. Early life bacterial diversity was inversely associated with hyperactivity/inattention at age 10 [bacterial OTUs (medium vs low: aOR = 0.4, 95%CI = (0.2–0.8)) and Chao1 (medium vs low: 0.3 (0.1–0.5); high vs low: 0.3 (0.2–0.6)], whereas fungal diversity was directly associated [Chao1 (high vs low: 2.1 (1.1–4.0)), Shannon (medium vs low: 2.8 (1.3–5.8)), and Simpson (medium vs low: 4.7 (2.4–9.3))]. At age 15, only Shannon index was significantly associated with hyperactivity/inattention [bacteria (medium vs low: 2.3 (1.2–4.2); fungi (high vs low: 0.5 (0.3–0.9))]. In conclusion, early life exposure to microbial diversity may play a role in the psychobehavioural development. We observe heterogeneity in the direction of the associations encouraging further longitudinal studies to deepen our understanding of the characteristics of the microbial community underlying the observed associations.

## Introduction

The worldwide prevalence of attention deficit and hyperactivity disorder (ADHD) is estimated to be close to 5%^1^. This neurodevelopmental disorder is characterized by inappropriate levels of hyperactivity, impulsivity and/or attention problems. Although genetics have shown to be one determinant in the development of ADHD^2^, environmental factors may also contribute to the development of this disorder^3–6^. One of such factors that has received little attention so far is the early life exposure to home microbial environment. Previous research has shown that the dust microbiota composition in early-life home is associated with the development of the immune system^7,8^ and several studies suggest a bidirectional relationship between the cognitive and the immunological development^9^. In addition, the home microbiota may influence the gut microbiota composition^10,11^, which also plays a role in the brain function and behavior^12–16^.

To our knowledge, only three studies have evaluated the associations of neuropsychological development with determinants of the indoor microbial environment^17–19^. They reported associations with observations of mold/dampness in the home, pet ownership and/or farm animal contact. However, the direction of the associations was heterogeneous (direct for farm animal contact and inverse for pet ownership and mold/dampness)^17–19^, and the observed effects were not confirmed by measurements of microbial markers in house dust^17^. Here, we present a case-cohort study nested in the German LISA (Lifestyle-related factors, Immune System and the development of Allergies in East and West Germany) birth cohort where we investigate the associations between early life exposure to microbial diversity in house dust and hyperactivity/inattention at the ages of 10 and 15 years.

## Methods

### Study design and population

The LISA study is a population based birth cohort where healthy full-term and normal birthweight neonates were recruited at birth in Munich, Leipzig, Wesel and Bad Honnef between 1997 and 1999 (n = 3094) and an informed consent was signed by their parents. Early life dust samples from bedroom floor were collected at the age of 3 months among children living in Munich and Leipzig (n = 2440). Questionnaires containing information on socio-demographic factors, environmental exposures and health were administered to the parents at their child’s birth, 3, 6, 12 and 18 months old, 2, 4, 6, 10, and 15 years old. At the ages of 10 and 15 years hyperactivity/inattention behavior was assessed using the Strengths and Difficulties Questionnaire (SDQ)^20,21^, administered to the parents at age 10 years and to the child at age 15 years. The study has been performed following the ethical principles described in the Declaration of Helsinki. The study was approved by the ethics committees of the Dep. of Medicine (Ludwig Maximillians University) and the Medical Faculty of the University of Leipzig at birth, the Medical Faculty of the University of Leipzig and the Bavarian Board of Physicians at the 10 year old follow-up and the Bavarian Board of Physicians and the Board of Physicians of Saxony at the 15 year old follow up. More detailed information is described elsewhere^3,22,23^.

**Figure 1:**
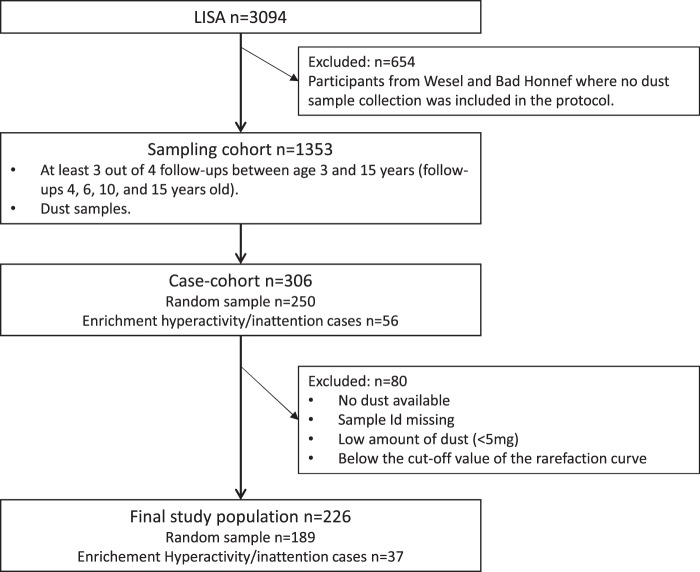
Selection of the study population.

Here, we present a case-cohort study^24^ nested in the LISA cohort. A description of the selection of the study population is provided in Fig. 1. The LISA sampling cohort comprises information on 1353 individuals with at least 3 out of 4 follow-ups between ages 4 and 15 years, and dust samples collected during early life. Children included in the sampling cohort had higher parental education, maternal age at birth and lower indoor smoking compared with the excluded. For the case-cohort design, we selected a random sample of 250 individuals out of the sampling cohort, comprising 16 hyperactivity/inattention cases at age 15 years. The random sample was enriched with all remaining cases of hyperactivity/inattention at the age of 15 years (n = 56). Eighty children were excluded because of no/low amounts of dust retrievable from the dust collection filters (<5 mg), no match between the child and the sample id, or samples producing insufficient number of sequence reads during amplicon sequencing. No statistically significant differences between included (n = 226) and excluded (n = 80) individuals were observed for the main variables considered. Season of dust sampling was significantly different in the two groups with higher percentage of samples collected during summer and lower during spring and autumn in the homes of not included children (see Table S1 in the Supplemental Material).

### Hyperactivity/inattention

Hyperactivity/inattention scores were assessed using the German parent-completed (at age 10 years) and self-completed (at age 15 years) versions of the SDQ^20,21,25,26^. As no published standards exist for Germans using the self-completed questionnaires at the 15 year, responses in the hyperactivity/inattention dimension of the SDQ at ages 10 and 15 years were categorized into hyperactivity/inattention symptoms yes/no according to recommended cut-offs for German populations for the parent-completed SDQ at age 10 years, being a child classified as hyperactivity/inattention when his/her score in the hyperactivity/inattention dimension was ≥7^26^. The agreement between the results of the hyperactivity/inattention dimension of the SDQ at ages 10 and 15 in the study population was low, with only 10 individuals scoring ≥7 in both tests.

### Dust sampling, sample processing, DNA extraction and sequence analysis

Dust samples were collected from the child’s bedroom floor at age 3 months using a vacuum cleaner supplied with an ALK filter holder^27^. All samples were stored at −20 °C, shipped on dry ice and processed and analyzed for microbial diversity in the National Institute for Health and Welfare (Kuopio, Finland). Dust was removed from the ALK filters, size homogenized, aliquoted, and stored at −20 °C. For DNA extraction, a target amount of 20 mg of dust was weighed into 2 mL glass-bead tubes; samples for which no or less than 5 mg of dust were retrievable were not considered in subsequent analyses. DNA was extracted as recently described in detail^28^, including a bead-milling step and clean-up with Chemagic DNA Plant–kit (PerkinElmer chemagen Technologie GmbG, Germany). Further detail on the sample processing is also provided in the Supplemental Material.

Bacterial 16S rRNA gene and fungal internal transcribed spacer region 1 (ITS1) PCR and amplicon sequencing were performed at a commercial sequencing partner LGC Genomics (Germany). Primers targeting the V4 region of the bacterial 16S rRNA gene (515F/806R) and the fungal ITS1 region (ITS1F/ITS2) primers were used^29,30^. Bacterial and fungal PCR’s amplicon sequencing and sequence processing were performed as earlier described^28^ with minor modifications (see details in the Supplemental Material). Processing and analyses of the 16S rRNA gene and ITS targeted amplicon reads relied largely on QIIME (Quantitative Insights Into Microbial Ecology) software version 1.9.1^31^, complemented by other software utilizing an in-house built analyses pipeline. Sequences were sorted with >97% similarity into operational taxonomic units (OTUs) using open reference OTU picking approach. Samples with less than 1026 sequences for bacteria and 996 sequences for fungi were excluded from the analysis; these same values were used as rarefaction value for calculation of alpha-diversity measures in QIIME, including observed OTU, Chao1, Simpson and Shannon indices. The first two are estimators of richness (i.e. the number of species in a community), while the Shannon and Simpson indices consider not only richness but also evenness (i.e. the homogeneity of abundance of individual OTUs in a sample). The Simpson index is less sensitive to richness and more to evenness than the Shannon index^32^.

### Potential confounders

Information on sex, study region, parental education, maternal age at birth, and having older siblings was obtained at birth. Parental education was defined as the highest education of mother or father and dichotomized as more than 10 years of education (“high”) and 10 years or less (“low”). Information on indoor factors or factors related to the dust sampling such as pet ownership, self-reports of mold or dampness in the home, indoor smoking, and date of sampling, was collected during the dust sampling, at birth or at the age of 6 months. Season of dust sampling was based on the date of sampling. Winter included December, January, and February; spring included March, April, and May; summer included June, July, and August; and fall included September, October, and November.

### Statistical analyses

Statistical analyses were performed using SAS (version 9.3; SAS Institute Inc., Cary, NC, USA) and R version 3.3.0 (R: A language and environment for statistical computing. R Foundation for Statistical Computing, Vienna, Austria). To compare the main characteristics and diversity measures of the study population we used Chi-squared test for categorical variables and Wilcoxon test for continuous variables. Spearman’s rank correlations was used to assess the relationship between the bacterial and fungal diversity measures. Since hyperactivity/inattention at age 10 was not considered in the definition of the study design, all statistical analyses including this health outcome were weighted. Individuals in the random sample received a weight equal to the inverse of the proportion of individuals in the random sample (n = 250) out of the total individuals in the cohort (n = 1353). In other words, they received a weight equal to 1/(250/1353) = 5.412. Individuals in the enrichment sample received a weight equal to 1. Generalized Additive Models (GAMs) were used to evaluate the functional relationship between the microbial diversity and hyperactivity/inattention. Since the shapes of the associations were generally not linear, we categorized the bacterial and fungal diversity indices using their respective tertiles (see cut-offs in Table S2 in the Supplemental Material). To evaluate the associations between the early life microbial diversity and hyperactivity/inattention, we used logistic regression models, obtaining odds ratios (OR) and their 95% confidence intervals (CI). For age 10, logistic regression models were weighted as described. For the age 15 models we used the “proc surveylogistic” SAS procedure that accounts for the study design and is recommended for samples without replacement from finite, or enumerable, populations. In sensitivity analyses, we ran unweighted logistic regression models for age 10 and age 15 symptoms including only individuals in the random sample.

## Results

A description of the characteristics of the study population and the microbial indices is provided in Table 1. Among the 226 included individuals, 84% (n = 189) were in the random sample. The prevalence of hyperactivity/inattention in the random sample at ages 10 and 15 years was 11% and 7%, respectively. The hyperactivity/inattention enrichment sample showed statistically significant lower medians for the fungal Simpson index. The four diversity measurements were strongly correlated between each other within both bacteria and fungi (Table 2). In contrast, the Shannon and Simpson indices were not correlated (data not shown), and the measures of richness were only weakly correlated [the number of observed OTUs (Spearman’s rho = 0.14) and Chao1 (Spearman’s rho = 0.33); p < 0.05], between bacteria and fungi.

**Table 1:** Description [n (%)] of the characteristics of the study population and early life indoor factors according to the study design.

	Study population	Random sample	Enrichment cases
			(hyperactivity/inattention age 15)
	N = 226	N = 189	N = 37
Socio-demographic factors			
Sex (girl)	109 (48.2)	94 (49.7)	15 (40.5)
Parental education (>10 years)	162 (72.0)	135 (71.8)	27 (73.0)
Study city (Munich)	146 (64.6)	118 (62.4)	28 (75.7)
Hyperactivity/inattention			
10 years old*	23 (11.7)	18 (10.9)	5 (16.1)
15 years old	50 (22.1)	**13 (6**.**9)**	**37 (100**.**0)**
Early life bedroom floor dust samples			
Microbial diversity measures (median and p25–p75)**			
Bacteria			
Number of observed OTUs	302 (254–366)	301 (253–369)	305 (267–341)
Chao1	767 (633–899)	761 (633–917)	789 (622–859)
Shannon	6.32 (5.81–7.01)	6.35 (5.78–7.10)	6.31 (5.94–6.65)
Simpson	0.95 (0.92–0.97)	0.95 (0.92–0.97)	0.94 (0.92–0.96)
Fungi			
Number of observed OTUs	164 (125–197)	167 (128–198)	149 (108–190)
Chao1	283 (235–353)	285 (235–360)	273 (236–313)
Shannon	5.22 (4.16–5.97)	5.31 (4.32–5.97)	4.74 (3.48–5.92)
Simpson	0.92 (0.83–0.96)	**0**.**93 (0**.**84–0**.**96)**	**0**.**89 (0**.**73–0**.**95)**
Season of dust sampling			
Winter	61 (27.0)	55 (29.3%)	6 (16.7%)
Spring	60 (26.6)	50 (26.6%)	10 (27.8%)
Summer	44 (19.5)	39 (20.7%)	4 (11.1%)
Autumn	61 (27.0)	44 (23.4%)	16 (44.4%)
Early life indoor factors			
Pet ownership	59 (26.2)	49 (26.2%)	9 (25.0%)
Siblings at birth	96 (42.5)	75 (39.9%)	20 (55.6%)
Indoor smoking	37 (16.4)	28 (14.9%)	9 (25.0%)
Mold at home	10 (4.4)	8 (4.3%)	2 (5.6%)

p25: 25^th^ percentile; p75:75^th^ percentile. Bold indicates p-value < 0.05 (Chi^2^ tests for categorical variables, and Wilcoxon tests for continuous variables). N total number of observations, n number of cases in each category, % percentage of characteristic in each category. *N = 196 (random sample N = 165; enrichment sample N = 31). **Bacteria: N = 224 (random N = 188; enrichment N = 36); fungi N = 218 (random N = 182; enrichment N = 36).

**Table 2:** Spearman’s rank correlation coefficients between the different diversity measures within bacteria and fungi.

	Observed OTUs	Chao1	Shannon	Simpson	
Observed OTUs		**0**.**94**	**0**.**92**	**0**.**73**	Bacteria
Chao1	*0*.*84*		**0**.**82**	**0**.**61**	
Shannon	*0*.*91*	*0*.*64*		**0**.**92**	
Simpson	*0*.*81*	*0*.*53*	*0*.*97*		
	*Fungi*				

The upper part is for bacteria (indicated in bold) and the lower part is for fungi (indicated in italics). All p-values are <0.05.

All medians of the bacterial diversity measures were lower among children with hyperactivity/inattention at age 10 and higher among children with hyperactivity/inattention at age 15, compared to those not presenting hyperactivity/inattention (Table 3). For fungi, no clear trend was observed for hyperactivity/inattention at age 10, but the four diversity measures were lower among children with hyperactivity/inattention at age 15. However, statistically significant difference was only observed for the fungal Simpson index.

**Table 3:** Description [median (p25-p75)] of the microbial diversity measures among children with and without hyperactivity/inattention at ages 10 and 15 years.

	Hyperactivity/inattention			
	10 years old		15 years old	
	yes	no	yes	no
Bacteria	n = 23	n = 171	n = 49	n = 175
Number of observed OTUs	280 (221–361)	302 (262–363)	309 (268–361)	300 (247–367)
Chao1	679 (544–845)	776 (635–893)	818 (673–876)	755 (624–914)
Shannon	6.17 (5.73–6.94)	6.29 (5.79–6.97)	6.32 (5.97–6.83)	6.29 (5.78–7.10)
Simpson	0.95 (0.93–0.97)	0.95 (0.92–0.97)	0.95 (0.93–0.97)	0.95 (0.92–0.97)
Fungi	n = 22	n = 168	n = 47	n = 171
Number of observed OTUs	163 (113–189)	165 (126–199)	150 (104–194)	167 (128–197)
Chao1	289 (191–357)	285 (236–356)	282 (233–341)	283 (235–357)
Shannon	5.24 (3.78–5.74)	5.16 (4.21–5.98)	4.85 (3.37–5.95)	5.31 (4.33–5.97)
Simpson	0.91 (0.78–0.94)	0.92 (0.83–0.96)	**0**.**89 (0**.**71–0**.**95)**	**0**.**93 (0**.**84–0**.**96)**

Bold indicates p-value < 0.05 (Wilcoxon test).

Descriptions of hyperactivity/inattention symptoms across tertiles of diversity indices and the results of the adjusted regression models are shown in Table 4. Bacterial richness (the number of observed OTUs and Chao1) was inversely associated, while fungal richness (Chao 1) and the evenness accounting diversity indices (Shannon and Simpson) were directly associated with hyperactivity/inattention at age 10. At age 15 years, only the Shannon index was significantly associated with hyperactivity/inattention, directly with bacteria (medium vs low) and inversely for fungi (high vs low). When we observe significant direct associations for medium vs low values the association shows an inverted U shape. In other words, when considering the medium tertile as reference, the ORs for age 10 and 15 for the high and low tertiles were generally below 1 (data not shown). ORs comparing high vs medium values were statistically significant for the bacterial Shannon and the fungal Simpson indices at ages 15 and 10, respectively (OR = 0.4; 95% CI: 0.2–0.7 and OR = 0.1; 95%CI: 0.05–0.3, respectively). In sensitivity analyses, we ran unweighted models including only the random sample (Table S3 in the Supplemental Material). The direction of the estimates was the same as shown in Table 4, and the effect sizes were similar. However, including only individuals in the random sample resulted in wide confidence intervals and loss of statistical significance in all cases except for the fungal Simpson index at age 10 years.

**Table 4:** Description (n and %) and adjusted associations (aOR and 95%CI) of hyperactivity/inattention at ages 10 and 15 years with early life diversity indices in tertiles.

	Hyperactivity/inattention			
	Age 10 years		Age 15 years	
	n (%)	aOR (95%CI)	n (%)	aOR (95%CI)
Bacteria	N = 194		N = 224	
Number of observed OTUs				
Low	10 (15.9%)	1	14 (18.9%)	1
Medium	5 (7.4%)	**0**.**41 (0**.**22–0**.**77)**	16 (21.9%)	1.27 (0.68–2.36)
High	8 (12.7%)	0.60 (0.32–1.12)	19 (24.7%)	1.30 (0.70–2.41)
Chao1				
Low	10 (15.6%)	1	12 (16.2%)	1
Medium	8 (12.1%)	**0**.**27 (0**.**14–0**.**51)**	19 (26.0%)	1.64 (0.89–3.01)
High	5 (7.8%)	**0**.**30 (0**.**16–0**.**57)**	18 (23.4%)	1.46 (0.76–2.81)
Shannon				
Low	7 (10.6%)	1	12 (16.2%)	1
Medium	9 (14.1%)	1.08 (0.61–1.93)	23 (31.5%)	**2**.**29 (1**.**24–4**.**23)**
High	7 (10.9%)	0.86 (0.47–1.58)	14 (18.2%)	0.92 (0.48–1.76)
Simpson				
Low	6 (9.0%)	1	15 (20.6%)	1
Medium	9 (13.9%)	0.65 (0.35–1.22)	21 (28.0%)	1.30 (0.72–2.32)
High	8 (12.9%)	1.21 (0.66–2.20)	13 (17.1%)	0.66 (0.36–1.22)
Fungi	N = 190		N = 218	
Number of observed OTUs				
Low	8 (12.5%)	1	19 (26.4%)	1
Medium	7 (12.1%)	1.34 (0.72–2.48)	13 (18.3%)	0.63 (0.34–1.16)
High	7 (10.3%)	1.02 (0.54–1.92)	15 (20.0%)	0.75 (0.41–1.40)
Chao1				
Low	7 (11.7%)	1	16 (22.2%)	1
Medium	7 (11.3%)	0.79 (0.41–1.55)	17 (23.6%)	0.93 (0.51–1.70)
High	8 (11.8%)	**2**.**13 (1**.**14–3**.**98)**	14 (18.9%)	0.80 (0.43–1.47)
Shannon				
Low	8 (12.5%)	1	21 (29.2%)	1
Medium	4 (10.3%)	**2**.**78 (1**.**34–5**.**77)**	10 (20.8%)	0.78 (0.40–1.50)
High	10 (11.5%)	1.64 (0.88–3.05)	16 (16.3%)	**0**.**51 (0**.**28–0**.**93)**
Simpson				
Low	8 (12.5%)	1	21 (29.2%)	1
Medium	10 (15.9%)	**4**.**74 (2**.**42–9**.**29)**	13 (18.1%)	0.65 (0.35–1.18)
High	4 (6.3%)	0.55 (0.25–1.22)	13 (17.6%)	0.57 (0.30–1.08)

Bold indicates p-value < 0.05. N total number of observations, n number of cases in each tertile, % percentage of cases in each tertile, aOR: adjusted odds ratio, 95%CI: 95% confidence interval. Adjusted for sex, parental education, city, siblings at birth, season of dust sampling, indoor smoking, pet ownership, and visible mold. Models for age 10 years are weighted (random sample weight = 5.412, enrichment sample weight = 1).

## Discussion

Our study suggests that the early life home indoor microbial environment may be associated with development of behavioral problems during childhood, however the direction of the associations observed is heterogeneous. Concerning hyperactivity/inattention at age 10, we show that exposure to high bacterial richness is associated with lower prevalence and high fungal richness with higher prevalence. Fungal Shannon and Simpson diversity indices show inverted U-shaped associations with hyperactivity/inattention at age 10. Regarding hyperactivity/inattention at age 15, significant associations are only observed for the Shannon diversity index, showing an inverted U-shaped association for bacteria and an inverse association for fungi.

A bidirectional relationship between the cognitive and the immunological development has been described^9^. Previous research has shown that the house dust microbiota may protect from developing asthma, but no significant associations have been reported for atopy^7,8^. Children with ADHD often present allergy or asthma symptoms^33–36^ and it has been hypothesized that both conditions may share common immunological mechanisms^37^. Therefore, it is possible that the house dust microbiota also plays a role in the development of ADHD through the early modulation of the immune system. Furthermore, the home environment during early life may also contribute to the development of the gut and lung microbiome^10,11^. Both gut and lung microbiome contribute to the development of the immune system^12,38,39^ and previous research has shown that the gut microbiota composition may be associated with ADHD^40–42^. However, the contribution of the house dust microbiome to the lung and gut microbiome is probably small and mechanisms involving direct interactions with the immune system may be more plausible.

In our study, bacterial and fungal richness (i.e. number of different taxa) was (inversely and directly, respectively) associated with hyperactivity/inattention at age 10 but not at age 15. We hypothesize that certain lifestyle factors that increase microbial richness could underlay our findings on hyperactivity/inattention during childhood and that the effect may be overlaid by other factors during adolescence. Frequent outdoor activities and/or occasional farm (animal) contact may contribute to an increase in bacterial richness and mold damage to fungal richness in house dust – via introduction of rare taxa –, more than to an increase in diversity indices assessed from the house dust microbiota^43^. This hypothesis is supported by previous research showing better cognitive development among children with occasional farm animal contact^17^, and by studies showing inverse associations between house mold/dampness and cognitive development, and positive associations with behavioral problems^17–19^.

Generally, bacterial and fungal Shannon and Simpson indices showed lower (non-significant) ORs for children in the highest tertiles as compared with the low tertile, showing inverted U-shape associations in most cases. Assuming the hypothesis that ADHD and asthma may share common mechanisms^37^, our results are in line with previous research showing protective effects of microbial diversity on asthma^7,44,45^. Moreover, the non-linear nature of the associations is in line with a previous research finding inverted U-shaped associations between asthma and microbial diversity scores^45^. Also, as shown in our recent publications on the association between early microbial exposures and asthma, it is possible that the observed association between diversity and ADHD is only a proxy of association between more specific microbial patterns or microbial activity^46,47^.

Among the potential limitations of our study, we should consider the assessment of hyperactivity/inattention. The SDQ is a screening questionnaire showing the perception of the parents (age 10) and the child (age 15) on behavioral problems, which does not exclude potential reporting bias. Nevertheless, it is considered a reliable and valid screening instrument^48,49^. Although studies assessing parent-child agreement on the SDQ reported moderate to high agreement for children with ADHD^50,51^, we observed low agreement^3^. This may be because the questionnaires were administered at two different time points and children presenting symptoms at age 10 may have received interventions to treat the symptoms. However, including reports of ADHD medication usage at age 15 did not result in higher agreements. Moreover, as no standards for child-administered SDQ in German population exist, for age 15 we used the standards defined for a German population for 10 year-old parent-administered SDQ^26^. Therefore, we cannot exclude the possibility that parent- and child- administered SDQ would require the use of different cut-offs to be comparable.

Another limitation that must be considered is the fact that dust samples from living-room floor were collected at one single time point, when children were three months old. In our study, we assumed that one single time point collection would be representative for the home microbial environment of the first months of life. Previous studies consistently showing associations between the indoor microbial environment during early life and the development of asthma, also collect dust samples at one time point^46,47^. Nevertheless, we cannot exclude the possibility that the indoor microbial environment changes across time and, therefore, our measures are only representative for the exposure during a short period of time.

The case-cohort design in our study has the advantage of maximizing the efficiency and reducing costs of a cohort study, allowing the investigation of additional study questions^24^. Nevertheless, this design involves a risk of losing representativeness of the random sample because of the censorings related with the definition of the sampling cohort. As reported in the methods section, our sampling cohort included children with higher parental education and differences in other related characteristics. Also, hyperactivity/inattention at age 10 was not considered in the study design. Moreover, a considerable proportion of house dust samples had to be excluded for various reasons (samples that could not be matched with a child, had no/low amount of dust (<5 mg), or yielded only low number of sequences not permitting sound diversity measures). This could have reduced statistical significance of the effect estimates. Nevertheless, it is important to note that our results were robust to the sensitivity analyses conducted including only the random sample.

Finally, an observational study cannot establish causality and we cannot exclude the possibility of residual confounding. Certain lifestyles or environmental exposures can modify the indoor microbial environment and have an impact on the development of behavioral problems. In our study, we had information on several potential confounders: pet ownership and mold^18,43,52^. Nevertheless, it is possible that other environmental exposures confound our results. For example, phthalates have been suggested to be associated with attention deficit disorder^53,54^, and microbial activity may contribute to phthalate degradation^55^. Unfortunately, we did not have information on phthalate exposure in our study. Previous research showed that phthalate exposure is associated with socioeconomic status^56^ and that may also be the case for other potential confounders. In our study, we adjusted our models for parental education as a proxy for socioeconomic status, thus we indirectly adjust for exposures associated with socioeconomic status.

Despite the acknowledged limitations, our study counts on a number of strengths that deserve to be mentioned. This case-cohort study is based on a prospective birth cohort where dust samples were collected during early life and the symptoms assessed 10 and 15 years later. This is the first study using actual next generation sequencing based measures of bacterial and fungal diversity in relation to behavioral problems during childhood and adolescence, rather than only determinants of the indoor microbial environment. In addition, we were able to adjust for many covariates that are related to hyperactivity/inattention and may determine the indoor microbial diversity.

## Conclusion

Our results suggest that early life microbial environment may play a role in the cognitive development, although the direction of the observed associations was heterogeneous. High numbers of different bacterial taxa present in house dust during the first months of life may reduce the risk of developing hyperactivity/inattention during childhood, the opposite may be the case for a rich presence of fungal taxa in house dust. Assessment of diversity scores that take into account the evenness of the microbial taxa rather than only their numbers, indicate that a more diverse fungal environment during early life may prevent from developing hyperactivity/inattention during adolescence, however, such associations with hyperactivity/inattention at age 15 for bacteria, as well as with hyperactivity/inattention at age 10 for fungi may not be linear. Larger studies are needed to understand the heterogeneous nature of our findings, further deepening into the characteristics of the microbial community factors that play a role in the associations observed.

## Supplementary information

Supplementary meterial
