# Supplementary material for: Early life home microbiome and hyperactivity/inattention in school-age children
Source: Sci Rep. 2019 Nov 22;9:17355. doi: 10.1038/s41598-019-53527-1 (PMC6874766; doi:10.1038/s41598-019-53527-1)
Supplement: Supplementary file 1 — Supplementary meterial [file 41598_2019_53527_MOESM1_ESM.doc]

**Early life home microbiome and hyperactivity/inattention in school-age children**

Lidia Casas, MD, PhD,^a^ Anne M. Karvonen, PhD,^b^ Pirkka V. Kirjavainen, PhD,^b,c^ Martin Täubel, PhD,^b^ Heidi Hyytiäinen, MSc,^b^ Balamuralikrishna Jayaprakash, MSc,^b^ Irina Lehmann, PhD,^d,e^ Marie Standl, PhD,^f^ Juha Pekkanen, MD, PhD* ^b,g^ and Joachim Heinrich, PhD*.^f,h^

*equal contribution.

**SUPPLEMENTAL MATERIAL**

**METHODS**

**Dust sampling and sample processing**

Early life (3 months old) dust samples were collected from the child´s bedroom floor using a vacuum cleaner supplied with an ALK filter holder containing a paper filter.^1^ The samples were stored at −20°C, shipped on dry ice and processed and analyzed for microbial diversity in the National Institute for Health and Welfare (THL, Kuopio, Finland). Filter cassettes were thawn at room temperature and processed within two hours. Dust was removed from the filters and samples were homogenized by sieving through a sterile strainer. Samples were aliquoted and stored at -20 °C until DNA extraction. For DNA extraction, a target amount of 20mg of dust was weighed into 2mL glass-bead tubes; samples where no or less than 5mg of dust were retrievable from the filters were not considered in subsequent analyses. DNA was extracted using the same protocol as recently described,^2^ including a bead-milling step and clean-up with Chemagic DNA Plant–kit (PerkinElmer chemagen Technologie GmbG, Germany).

**Bacterial and fungal amplicon sequencing and bioinformatics processing**

Bacterial 16S rRNA gene and fungal internal transcribed spacer region 1 (ITS1) PCR and amplicon sequencing were performed at a commercial sequencing partner LGC Genomics (Germany). Primers targeting the V4 region of the bacterial 16S rRNA gene (515F/806R) and the fungal ITS1 region (ITS1F/ITS2) primers were used.^3,4^ A detailed description of the PCR protocol, amplicon sequencing and sequence processing as performed – with minor modifications - in this current study is provided elsewhere.^2^ Rather than a nested PCR approach, direct PCR without prior pre-amplification was performed, applying 30 and 35 cycles in the 16S rRNA gene and ITS1 region amplification, respectively, using the primers specified above and PCR conditions specified in the earlier paper. Sequencing was performed on an Illumina MiSeq with V3 chemistry resulting in paired-end reads with a length of 300 bp each. The libraries were demultiplexed using Illumina's bcl2fastq v1.8.4 (https://support.illumina.com/downloads/bcl2fastq_conversion_software_184.html) and all sequence reads processed with custom Python v2.7.6 scripts to sort them by sample, removing barcode and amplicon primers sequences. Adapter sequences were removed from the 3' end of reads with a proprietary script discarding reads shorter than 100 bp.

Processing and analyses of the 16S rRNA gene and ITS targeted amplicon reads relied largely on QIIME (Quantitative Insights Into Microbial Ecology) software version 1.9.1, complemented by other software utilizing an in-house built analyses pipeline as described.^5^ The raw bacterial reads were preprocessed by removal of artificial sequences such as adapters by cutadapt software version 1.14,^6^ followed by trimming of bad quality reads and ambiguous sequences by the Trimmomatic software version 0.35.^7^ Then, the preprocessed reads were merged using FLASH2 (Fast Length Adjustment of SHort reads) software.^8^ UCHIME^9^ was employed to remove chimeras in the preprocessed reads using the VSEARCH version 2.5.2.^10^ After chimera removal, the preprocessed reads were aligned using pynast^11^ with greengenes database gg_13_8 release^12^ in the case of bacteria, and UNITE database version7_dynamic_28.06.2017 release^13^ in the case of fungi. Reads were sorted with >97% similarity into operational taxonomic units (OTUs) using open reference OTU picking approach. OTUs representing less than 0.001% of the total sequences were excluded from the bacterial OTU table, as were chloroplast and mitochondrial sequences. The bacterial sequence data were affected by a *Ralstonia* spp. contamination that occurred during PCR at the sequencing provider. Due to the inclusion of all necessary controls and the specificity of the contamination, this issue was solved by excluding this one specific *Ralstonia* OTU from the bacterial dataset.

Samples with less than 1026 sequences for bacteria and at 996 sequences for fungi were excluded from the analysis; these same values were used as rarefaction value for calculation of alpha-diversity measures in QIIME, including the number of observed OTU and Chao1, Simpson and Shannon diversity indices. The first two measures are estimators of richness (i.e. the number of species in a community), while the Shannon and Simpson indices consider not only richness but also evenness (i.e. the homogeneity of relative abundance of individual OTUs in a sample). Simpson index is less sensitive to richness and more to evenness than Shannon index, and Shannon index is more sensitive to evenness than the number of observed OTUs or the Chao1 index.^14^

**TABLES**

Table S1. Description [n (%)] of the socio-demographic characteristics, hyperactivity/inattention symptoms and indoor factors among included and not included study population due to availability of microbial measurements.

|  |  |  |  | Included | Not included |
| --- | --- | --- | --- | --- | --- |
|  |  |  |  | N=226 | N=80 |
| Socio-demographic factors | | | |  |  |
|  | Sex (girl) | | | 109 (48.2%) | 37 (46.3%) |
|  | Parental education (> 10 years) | | | 162 (72.0%) | 56 (70.9%) |
|  | Study city (Munich) | | | 146 (64.6%) | 55 (68.8%) |
| Hyperactivity/inattention | | | |  |  |
|  |  | 10 years old* | | 23 (11.7%) | 9 (13.0%) |
|  |  | 15 years old | | 50 (22.1%) | 22 (27.5%) |
|  | Season of dust sampling | | |  |  |
|  |  | Winter | | **61 (27.0%)** | **20 (25.0%)** |
|  |  | Spring | | **60 (26.6%)** | **15 (18.8%)** |
|  |  | Summer | | **44 (19.5%)** | **29 (36.3%)** |
|  |  | Autumn | | **61 (27.0%)** | **16 (20.0%)** |
|  | Pet ownership | | | 59 (26.2%) | 24 (30.0%) |
|  | Siblings at birth | | | 96 (42.5%) | 37 (46.3%) |
|  | Indoor smoking | | | 37 (16.4%) | 11 (13.8%) |
|  | Mold at home | | | 10 (4.4%) | 4 (5.0%) |

Bold indicates p-value<0.05 in chi square tests. N total number of observations, n number of cases in each category, % percentage of characteristic in each category.
*Included N=196; not included N=69.

Table S2. Cut-offs used for the categorization of the diversity measures in tertiles.

|  | Bacteria |  | Fungi |  |
| --- | --- | --- | --- | --- |
|  | p33 (low) | p66 (high) | p33 (low) | p66 (high) |
| Number of observed OTUs | 270.30 | 336.40 | 136.90 | 185.10 |
| Chao1 | 665.93 | 857.32 | 254.59 | 322.31 |
| Shannon | 5.96 | 6.70 | 4.59 | 5.74 |
| Simpson | 0.93 | 0.97 | 0.87 | 0.95 |

Table S3. Adjusted* associations (aOR and 95%CI) of hyperactivity/inattention at ages 10 and 15 with early life diversity indices in tertiles including only individuals in the random sample.

|  |  |  | Hyperactivity inattention | |
| --- | --- | --- | --- | --- |
|  |  |  | Age 10 years | Age 15 years |
| Bacteria | | | N=164 | N=188 |
|  | Number of observed OTUs | |  |  |
|  |  | Low | 1 | 1 |
|  |  | Medium | 0.44 (0.10-1.94) | 1.38 (0.17-11.18) |
|  |  | High | 0.60 (0.14-2.68) | 6.00 (0.94-38.15) |
|  | Chao1 | |  |  |
|  |  | Low | 1 | 1 |
|  |  | Medium | 0.25 (0.05-1.16) | 1.25 (0.16-9.50) |
|  |  | High | 0.29 (0.06-1.31) | 5.03 (0.82-30.72) |
|  | Shannon | |  |  |
|  |  | Low | 1 | 1 |
|  |  | Medium | 1.00 (0.25-3.95) | 0.81 (0.13-5.14) |
|  |  | High | 0.81 (0.20-3.33) | 2.58 (0.54-12.33) |
|  | Simpson | |  |  |
|  |  | Low | 1 | 1 |
|  |  | Medium | 0.61 (0.14-2.71) | 0.71 (0.12-4.13) |
|  |  | High | 1.12 (0.27-4.71) | 1.54 (0.33-7.18) |
| Fungi | | | n=159 | n=182 |
|  | Number of observed OTUs | |  |  |
|  |  | Low | 1 | 1 |
|  |  | Medium | 1.46 (0.33-6.47) | 0.30 (0.04-2.20) |
|  |  | High | 1.08 (0.24-4.96) | 1.34 (0.27-6.66) |
|  | Chao1 | |  |  |
|  |  | Low | 1 | 1 |
|  |  | Medium | 0.80 (0.16-4.04) | 0.44 (0.05-3.64) |
|  |  | High | 2.23 (0.50-9.95) | 2.39 (0.45-12.69) |
|  | Shannon | |  |  |
|  |  | Low | 1 | 1 |
|  |  | Medium | 3.63 (0.59-22.16) | 0.39 (0.04-4.16) |
|  |  | High | 1.94 (0.41-9.08) | 1.01 (0.22-4.75) |
|  | Simpson | |  |  |
|  |  | Low | 1 | 1 |
|  |  | Medium | **6.14 (1.12-33.65)** | 0.89 (0.16-4.94) |
|  |  | High | 0.65 (0.09-4.64) | 0.82 (0.15-4.67) |

Bold indicates p-value<0.05. N total number of observations, aOR: adjusted odds ratio, 95%CI: 95% confidence interval.
*Adjusted for sex, parental education, city, siblings at birth, season of dust sampling, indoor smoking, pet ownership, and visible mold.

**REFERENCES**

1. Casas L, Tischer C, Wouters IM, et al. Endotoxin, extracellular polysaccharides, and β(1-3)-glucan concentrations in dust and their determinants in four European birth cohorts: results from the HITEA project. *Indoor Air*. 2013;23(3):208-218. doi:10.1111/ina.12017.

2. Jayaprakash B, Adams RI, Kirjavainen P, et al. Indoor microbiota in severely moisture damaged homes and the impact of interventions. *Microbiome*. 2017;5(1):138. doi:10.1186/s40168-017-0356-5.

3. Caporaso JG, Lauber CL, Walters WA, et al. Global patterns of 16S rRNA diversity at a depth of millions of sequences per sample. *Proc Natl Acad Sci U S A*. 2011;108 Suppl 1(Supplement_1):4516-4522. doi:10.1073/pnas.1000080107.

4. Smith DP, Peay KG. Sequence depth, not PCR replication, improves ecological inference from next generation DNA sequencing. *PLoS One*. 2014;9(2):e90234. doi:10.1371/journal.pone.0090234.

5. Caporaso JG, Kuczynski J, Stombaugh J, et al. QIIME allows analysis of high-throughput community sequencing data. *Nat Methods*. 2010;7(5):335-336. doi:10.1038/nmeth.f.303.

6. Martin M. Cutadapt removes adapter sequences from high-throughput sequencing reads. *EMBnet.journal*. 2011;17(1):10. doi:10.14806/ej.17.1.200.

7. Bolger AM, Lohse M, Usadel B. Trimmomatic: a flexible trimmer for Illumina sequence data. *Bioinformatics*. 2014;30(15):2114-2120. doi:10.1093/bioinformatics/btu170.

8. Magoč T, Salzberg SL. FLASH: fast length adjustment of short reads to improve genome assemblies. *Bioinformatics*. 2011;27(21):2957-2963. doi:10.1093/bioinformatics/btr507.

9. Edgar RC, Haas BJ, Clemente JC, Quince C, Knight R. UCHIME improves sensitivity and speed of chimera detection. *Bioinformatics*. 2011;27(16):2194-2200. doi:10.1093/bioinformatics/btr381.

10. Rognes T, Flouri T, Nichols B, Quince C, Mahé F. VSEARCH: a versatile open source tool for metagenomics. *PeerJ*. 2016;4:e2584. doi:10.7717/peerj.2584.

11. Caporaso JG, Bittinger K, Bushman FD, DeSantis TZ, Andersen GL, Knight R. PyNAST: a flexible tool for aligning sequences to a template alignment. *Bioinformatics*. 2010;26(2):266-267. doi:10.1093/bioinformatics/btp636.

12. DeSantis TZ, Hugenholtz P, Larsen N, et al. Greengenes, a chimera-checked 16S rRNA gene database and workbench compatible with ARB. *Appl Environ Microbiol*. 2006;72(7):5069-5072. doi:10.1128/AEM.03006-05.

13. Abarenkov K, Henrik Nilsson R, Larsson K-H, et al. The UNITE database for molecular identification of fungi--recent updates and future perspectives. *New Phytol*. 2010;186(2):281-285. doi:10.1111/j.1469-8137.2009.03160.x.

14. Colwell RK. Biodiversity: Concepts, patterns, and measurement. In: Levin SAS, ed. *The Princeton Guide to Ecology*. Princeton, NJ: Princeton University Press; 2009:257-263.
